# Toast

**Published:** 1855-10

**Authors:** 


*Toast.*—The following poetical toast, by Dr. Oliver Wendell Holmes, was prepared in the expectation that there would be a public dinner at the late meeting of the American Medical Association, and with the intention that it should be offered on that occasion:—

A triple health to Friendship, Science, Art,
From heads and hands that own a common heart!
Each in its turn the other's willing slave:
Each in its season strong to heal and save.

Friendship's blind service, in the hour of need,
Wipes the pale face—and lets the victim bleed.
Science must stop to reason and explain:
Art claps his fingers on the streaming vein.

But Art's brief memory fails the hand at last;
Then Science lifts the flambeau of the past.
When both their equal impotence deplore—
When Learning sighs, and Skill can do no more,
The tear of Friendship pours its heavenly balm,
And soothes the pang no anodyne may calm!

